# Miliary pattern of brain metastases – a case report of a hyperacute onset in a patient with malignant melanoma documented by magnetic resonance imaging

**DOI:** 10.1186/s13014-015-0459-8

**Published:** 2015-07-19

**Authors:** Florian P. Reiter, Clemens Giessen-Jung, Mario M. Dorostkar, Birgit Ertl-Wagner, Gerald U. Denk, Suzette Heck, Christina T. Rieger, Hans W. Pfister, Mark op den Winkel

**Affiliations:** Department of Internal Medicine II, Liver Center Munich, University of Munich, Grosshadern Campus, Marchioninistr. 15, D-81377 Munich, Germany; Department of Internal Medicine III, Hematology and Medical Oncology, University of Munich, Grosshadern Campus, Marchioninistr. 15, D-81377 Munich, Germany; Center for Neuropathology and Prion Research, University of Munich, Feodor-Lynen-Str. 23, D-81377 Munich, Germany; Institute for Clinical Radiology, University of Munich, Grosshadern Campus, Marchioninistr. 15, D-81377 Munich, Germany; Department of Neurology, University of Munich, Grosshadern Campus, Marchioninistr. 15, D-81377 Munich, Germany

**Keywords:** Melanoma, Multiple melanoma metastases, Brain metastases

## Abstract

**Background:**

Miliary brain metastases are a rare condition but associated with an exceedingly poor prognosis. We present the case of a patient suffering from malignant melanoma with an acute progressively worsening of neurological symptoms up to the loss of consciousness. The magnetic resonance imaging (MRI) demonstrated a new onset of disseminated, miliary spread of central nervous system metastases from a malignant melanoma within 4 days.

**Case presentation:**

We report on a 57-year-old woman suffering from metastatic malignant melanoma positive for BRAF-V600E mutation who developed an acute onset of neurological symptoms. The patient received vemurafenib and dacarbacin as chemotherapeutic regime for treatment of malignant melanoma. After admission to our hospital due to progressive disturbance of memory and speech difficulty a magnetic resonance tomography (MRI) was performed. This showed no evidence of cerebral tumour manifestation. The symptoms progressed until a loss of consciousness occurred on day five after admission and the patient was admitted to our intensive care unit for orotracheal intubation. No evidence for infectious, metabolic or autoimmune cerebral disorders was found. Due to the inexplicable acute worsening of the neurological symptoms a second MRI was performed on day five. This revealed a new onset of innumerable contrast-enhancing miliary lesions, especially in the grey matter which was proven as metastases from malignant melanoma on histopathology.

**Conclusion:**

This case describes an unique hyperacute onset of tumour progression correlating with an acute deterioration of neurological symptoms in a patient suffering from miliary brain metastasis from BRAF positive malignant melanoma.

## Background

The incidence of malignant melanoma is increasing during the last decades, especially in the Caucasian population [1]. Cutaneous Melanoma reflects the skin cancer with the highest mortality [2]. Malignant melanoma commonly metastasizes to the central nervous system (CNS) [3] and cerebral metastases from malignant melanoma are known to have a variety of appearances and cerebral locations [4]. In general the brain is a frequent site of metastasis in patients suffering from disseminated malignant melanoma [5] however only 5 % of patients with multiple melanoma metastases have more than five intracerebral metastatic lesions [3], and especially a miliary pattern seems to be a rarity [3, 6]. A miliary pattern of brain metastases refers to a widespread dissemination of innumerable small punctate lesions. This condition indicates an exceedingly poor prognosis [3].

We present the case of a 57-year old female patient with an acute progressively worsening of neurological symptoms up to the loss of consciousness. The magnetic resonance imaging (MRI) demonstrated a new onset of disseminated, miliary spread of CNS metastases from a malignant melanoma within 4 days. The diagnosis was proven by histopathology.

## Case presentation

A 57-year old woman was admitted to our hospital with a 3-day history of progressive disturbance of memory and speech difficulty. The patient was suffering from known pulmonary, hepatic, subcutaneous and bone metastases 21 months after the initial diagnosis and excision of a malignant melanoma on the helix of the right auricle (Clark Level 2, BRAF status: V600E positive). Metastatic disease was histologically confirmed in a cutaneous biopsy at this point. The patient had been treated with a chemotherapeutic regimen with vemurafenib 960 mg and dacarbacin 1000 mg/m^2^ for 2 months prior to admission.

Six months after the initial diagnosis of malignant melanoma, the patient had additionally been diagnosed with a neuroendocrine tumour (NET) of the terminal ileum with a Ki-67 index of 8–10 %. The patient subsequently underwent a laparoscopic resection of the ileocecal region.

One day after admission, the patient had signs of an expressive aphasia, paraphasic errors, word substitution errors, and impaired repetition. She had difficulties in understanding conversation, but she was able to follow simple commands, recall was poor. An impaired coordination with tremor was present. The patient was oriented regarding person but only partially regarding time and situation. No cranial nerve dysfunction, pareses or pyramidal tract signs were present. Deep tendon reflexes were adequate on both sides. Sensibility was not impaired.

One day after admission, a gadolinium enhanced MRI of the brain was performed. Neither cerebral metastases nor signs of meningeal carcinomatosis were noted. A lumbar puncture was subsequently performed. Cerebrospinal fluid (CSF) analysis showed no detectable leukocytes, an elevated total protein content (54 mg/dl) and a normal CSF glucose (69 mg/dl). Oligoclonal IgG bands were negative in serum and CSF. No malignant cells were detected in the CSF by microscopy.

An antiepileptic therapy with benzodiazepines (lorazepam) and levetiracetam was initiated, which did not lead to an improvement of the clinical situation. An EEG did not reveal signs of nonconvulsive status epilepticus.

Subsequently, a contrast enhanced CT scan of the neurocranium was obtained 2 days after admission. This was essentially normal without signs of haemorrhage, ischemia, metastases or elevated intracranial pressure. During the following 4 days, the patient´s level of consciousness progressively decreased and the patient became somnolent. At this point the patient was aphasic with completely absent speech production. The patient was unable to follow instructions. The Babinski sign was absent and deep tendon reflexes were unremarkable. Metabolic or endocrinological causes for the patient´s somnolence were excluded.

The patient was admitted to our intensive care unit for orotracheal intubation on day 5 after admission due to a Glasgow Coma Scale score of 7.

**Fig. 1:**
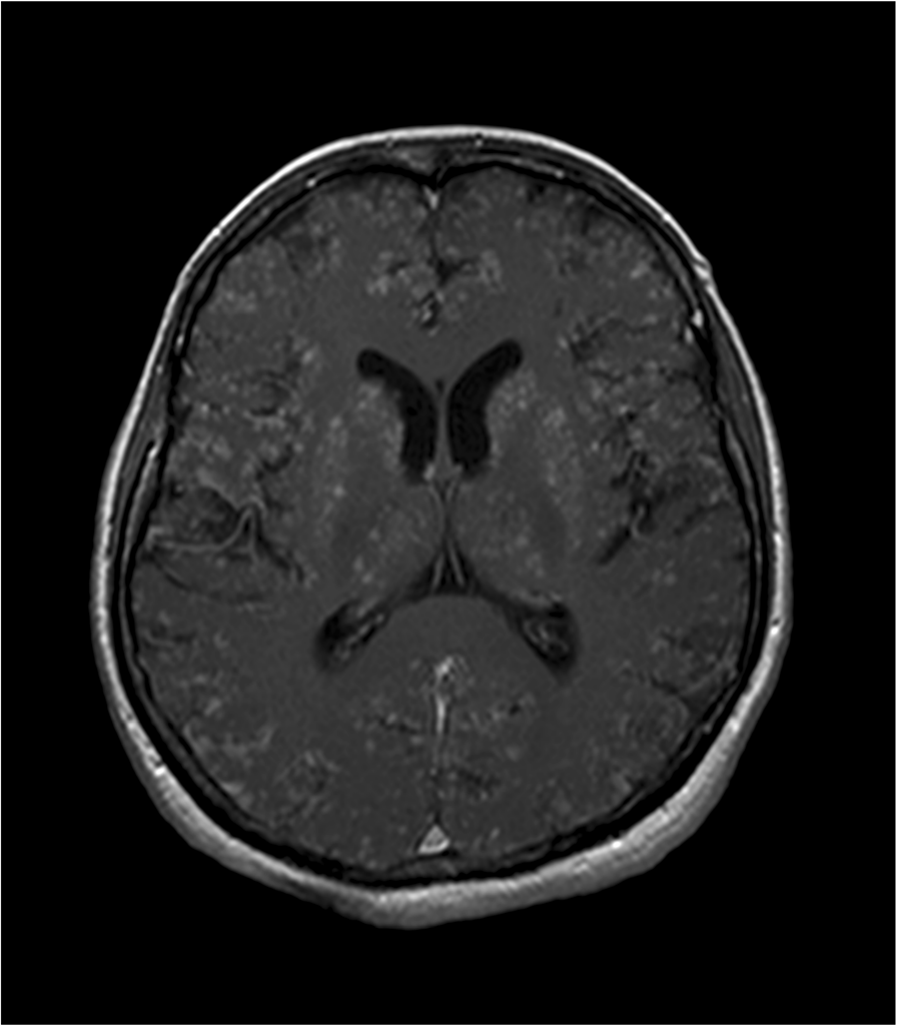
T1-weighted sequence of MRI. The axial T1-weighted sequence after intravenous administration of a Gadolinium-based contrast medium demonstrates a military pattern of innumerable small contrast-enhancing lesions with a predilection of the grey matter including the cerebral cortex, basal ganglia and thalami

**Fig. 2:**
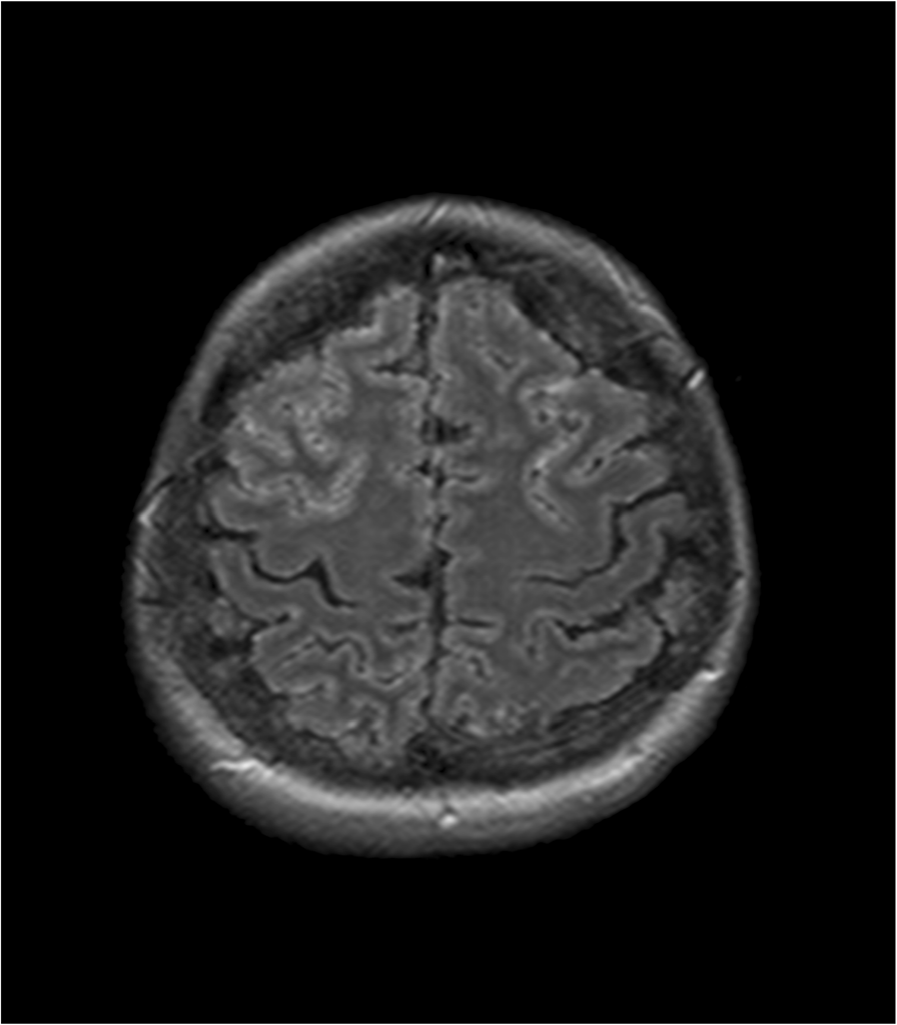
FLAIR sequence of MRI. The axial FLAIR sequence shows multiple small areas of signal hyperintensities in the grey matter and in the leptomeninges

On day 5 after admission, a further gadolinium enhanced MRI of the brain was performed, which now demonstrated innumerable contrast-enhancing miliary lesions, especially in the grey matter of the cerebrum, cerebellum, putamen, caudate nucleus and thalamus accompanied by low grade edema (Figs. 1 and 2). Repeat review of the initial MRI again confirmed the absence of these lesions at that time.

A repeated lumbar puncture again showed no leukocytes in the CSF, an elevated total protein content (73 mg/dl), a normal CSF-serum glucose ratio (86/165 mg/dl) and again, oligoclonal IgG bands were negative in serum and CSF. No malignant cells were detected in the CSF by microscopy.

**Fig. 3:**
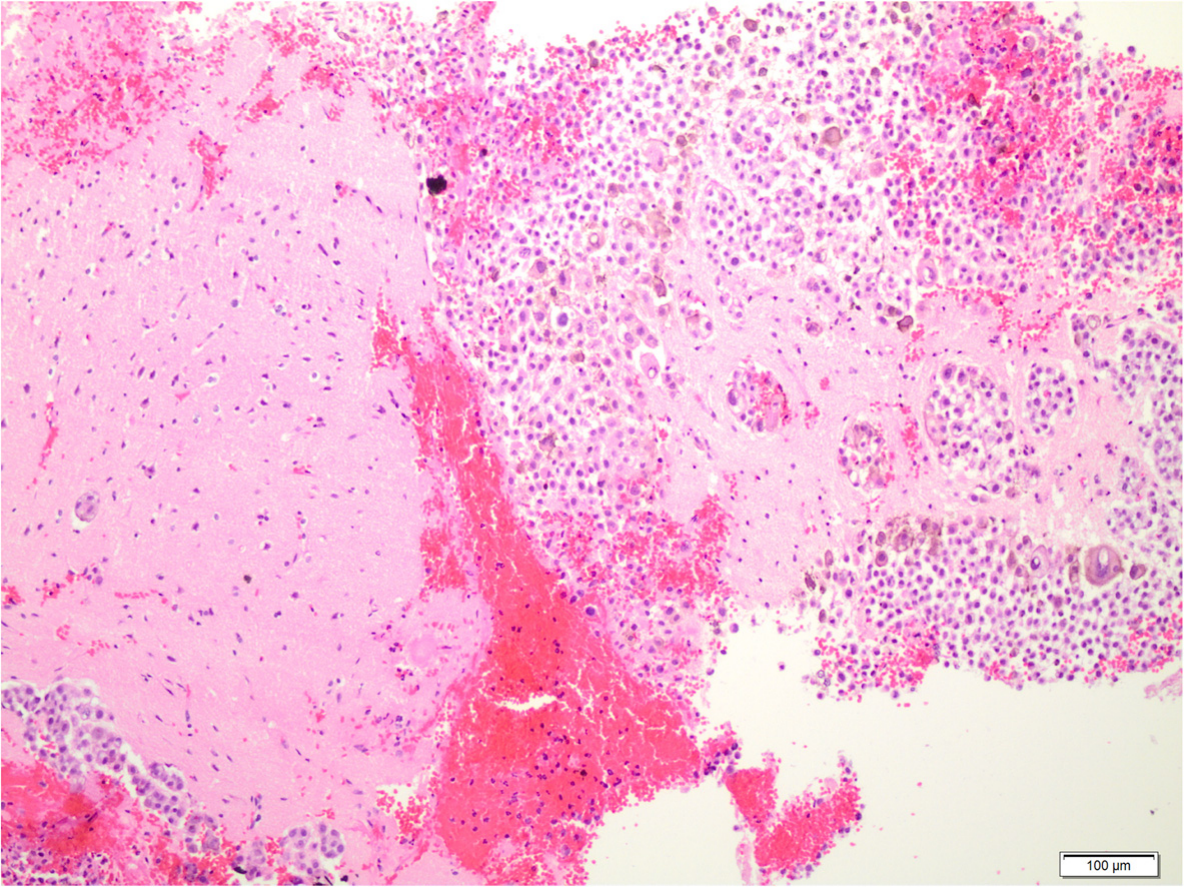
H&E stain. H&E stain of a biopsy from a suspect cortical lesion, showing invasion of the cortex with numerous tumour cells. Some tumour cells bear conspicuous brownish pigment

**Fig. 4:**
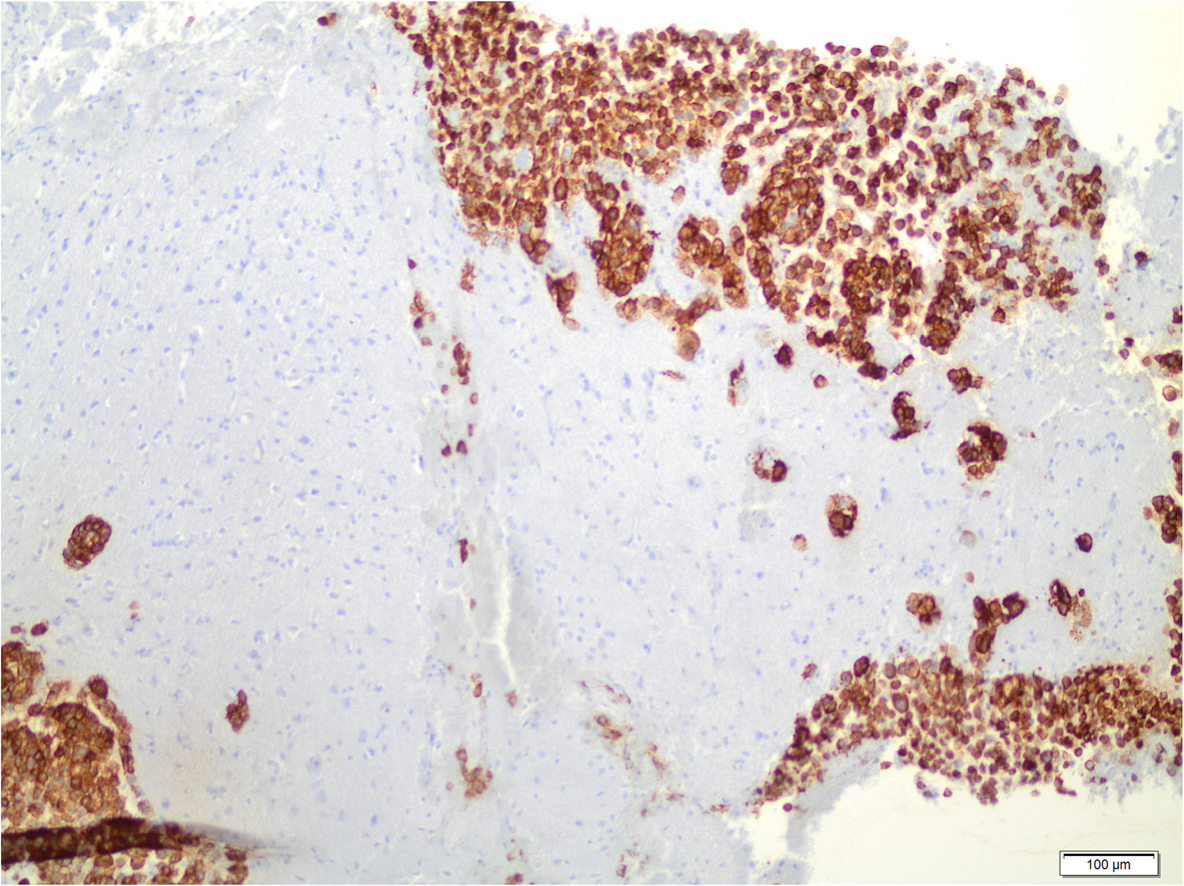
HMB-45 stain. HMB-45 stain of the biopsy sample shown in Fig. 4, showing positive immunoreactivity, verifying melanoma origin

Due to the uncommon presentation of the acute-onset miliary pattern in the cerebral grey matter, brain biopsies of the frontal right surficial cortex area were performed to exclude infectious and autoimmune causes and to potentially differentiate the type of metastases. Intraoperative inspection demonstrated dark spotted lesions disseminated over the meninges and the cerebrum. Histologic examination confirmed cerebral metastases of a malignant melanoma (Figs. 3 and 4). The molecular examination of the brain metastasis confirmed BRAF mutation also in this compartment.

Due to the rapidly deteriorating condition and a dismal prognosis, after interdisciplinary discussion with oncologists, dermatologists and radiation oncologists it was decided to extubate the spontaneously breathing patient. She received palliative care and deceased 5 days after the biopsy without regaining consciousness.

Our patient experienced a very acute onset of clinically relevant cerebral metastases within 4 days from the initial MRI of the brain, which had not shown evidence of cerebral metastases. The demonstration of miliary metastases in the second MRI of the brain was accompanied by an exacerbation of neurological symptoms. The acute deterioration of the disease process occurred 23 months after the initial diagnosis of malignant melanoma under active treatment with vemurafenib 960 mg and dacarbacin 1000 mg/m^2^ for the past two months.

Vemurafenib is an oral tyrosine kinase inhibitor targeting the oncogenic BRAF V600 protein kinase. It is approved for the treatment of BRAF V600 mutation-positive unresectable or metastatic melanoma [7]. Despite improved response rates and increased survival, malignant melanoma cells can acquire resistance to B-RAF (V600E) by receptor tyrosine kinase-mediated activation of alternative pathways [8]. In our case, the rapid progression under targeted therapy for metastatic melanoma is suggestive of an accelerated alternative pathway activation and massive cerebral spread of the disease being associated with severe worsening of neurologic symptoms.

Infections of the nervous system can cause contrast-enhanced lesions [9]. In our case the patient showed a normal cell count with no detectable leukocytes and a normal glucose level within the CSF, which makes CNS-listeriosis or bacterial meningitis unlikely. Cryptococcal disease was not detected in culture or antigen test. Subdural and intracerebral swab was negative for tuberculosis. No evidence for sarcoidosis was found within the CSF or computed tomography of the thorax. Due to an involvement of the grey matter an acute disseminated encephalomyelitis can be excluded.

Bearing in mind the two different malignant entities in the patient’s recent medical history, histological examination of the brain lesions was mandatory. NETs are the origin of brain metastases in approximately 1.4 % of all patients with cerebral metastases [10]. In patients with NET, the incidence of brain metastases is estimated to be up to 5 % [10]. Nevertheless, considering the fact that most NET brain metastases have their origin in the bronchopulmonary tract [10] and that the patients NET of the ileum had a low Ki-67 index of 8–10 %, the pretest-probability of a positive histology for NET especially in a patient with known melanoma metastases in other parts of the body was low. Furthermore to our best knowledge, as opposed to melanoma no case of miliary brain metastases of a NET has been reported, so far. Miliary brain metastases were reported several times in patients suffering from lung cancer [11–13]. These cases describe a slow onset of symptoms. Further, it is reported that the cerebral lesions may not be detected in computed tomography as seen in our case [13]. The study by Rivas et al. described a rapidly onset of dementia in a patient suffering from miliary brain metastases of unknown primary adenocarcinoma [14]. This patient deceased five and a half months after onset. Taken together none of the mentioned studies showed a comparable hyperacute onset as seen in our case.

To our best knowledge no association between BRAF mutations and multiple organ metastases were investigated so far. The fact that the molecular analysis of the brain metastases in our case confirmed a BRAF mutation in this tissue raises the question whether there could be also a correlation of the BRAF status with multiple and miliary organ metastasis as seen under EGRF mutations in lung cancer [15–18].

## Conclusion

Since there was no evidence of cerebral metastasis in the initial MRI this report describes a very rapid tumour progression in a patient suffering from malignant melanoma that timely correlated with an acute worsening of consciousness and neurological symptoms.

Thereby it illustrates an unique time course of disease progression in metastatic malignant melanoma.

Furthermore this case confirms the fact that malignant melanoma has the potential to cause miliary brain metastasis and raises the question if there might be an association between BRAF status and miliary pattern of metastasis.

## Consent

Since the patient was unable to provide consent, the written informed consent for publication of this case report and any accompanying images was obtained from the son of the patient. A copy of the written consent is available for review by the Editor-in-Chief of this journal.
